# How culture shapes subjective cognitive decline reporting: Refining assessment tools with digital solutions

**DOI:** 10.1002/alz.71141

**Published:** 2026-01-24

**Authors:** Rong Sun, Deqi Kong

**Affiliations:** ^1^ Affiliated Hospital of Nanjing University of Chinese Medicine Nanjing China; ^2^ Center for Disease Control and Prevention of Wu Zhong District Suzhou China

**Keywords:** Alzheimer's disease, cognitive assessment, cultural influences, digital biomarkers, subjective cognitive decline

1

To the Editor:

We read with great interest the study by Farias et al.^1^ titled, “Subjective cognitive decline among diverse older adults: Prevalence and associations with objective cognition.” Their findings highlight important ethnic disparities in subjective cognitive decline (SCD) reporting and its association with objective cognitive performance, emphasizing the critical need to incorporate cultural considerations into cognitive assessment. Building upon this work, we would like to discuss several methodological considerations and explore how integrating insights from neuropsychology and digital health could enhance the clinical utility of SCD for early dementia detection and prevention.

The observed dissociation between SCD reports and objective cognitive performance, particularly among Hispanic/Latinx participants, strongly suggests profound cultural influences on the perception and reporting of cognitive concerns. Cultural factors, including societal norms regarding aging and memory, explanatory models of cognitive changes (e.g., attributing them to stress or normal aging rather than underlying pathology), the stigma associated with cognitive impairment, and communication styles likely shape how individuals interpret and report cognitive experiences.^2^ These influences extend across various cognitive domains. For instance, cultural emphasis on collective versus individual memory may affect the reporting of autobiographical details, while norms regarding communication styles could influence how changes in language fluency are perceived. This necessitates culturally sensitive public health messaging and clinical assessment approaches. Similarly, clinicians require training and tools to effectively navigate these cultural differences, ensuring reported concerns are interpreted within appropriate cultural frameworks to avoid under‐ or overexamination. This cultural perspective is essential for translating SCD research into equitable clinical practice and community outreach. Public health initiatives could benefit from culturally adapted cognitive screening tools that account for these factors while maintaining sensitivity to true neurodegenerative risk.

While the Everyday Cognition scale 12 item (ECog‐12) demonstrates good psychometric properties, its dichotomous operationalization of SCD (endorsement of any item as “consistently worse”) may obscure important clinical information. This approach, while practical, fails to capture the continuum of symptom severity, particularly in sensitive domains like executive function, in which this simplification may attenuate associations with objective cognitive performance. More critically, the measure may not adequately assess culturally specific cognitive domains (e.g., collective decision making valued in non‐Western cultures), potentially leading to the mischaracterization of SCD in minority populations. Beyond ECog‐12, cultural interpretation similarly affects responses to other commonly used SCD instruments (e.g., Subjective Cognitive Decline Questionnaire [SCD‐Q], Memory Complaint Questionnaire [MAC‐Q]), indicating that measurement bias may be widespread across self‐report frameworks rather than confined to a single questionnaire.

The study's use of composite cognitive scores, while improving statistical power, presents several limitations. For executive function, combining distinct subdomains (working memory, mental flexibility) into a single composite may mask differential relationships with SCD. More importantly, standardization using full cohort means may artificially create or amplify ethnic differences if systematic variations in baseline cognitive performance exist across groups due to factors like education disparities.

The heterogeneity of SCD‐related factors underscores the need for more precise, multidimensional assessment beyond simple prevalence rates or global scores. Traditional questionnaires like the ECog are limited by their reliance on culturally mediated self‐perception. Artificial intelligence (AI)–derived digital biomarkers, particularly the automated analysis of spontaneous speech (natural language processing [NLP]), offer promising alternatives. Fraser et al.^3^ demonstrated that NLP features extracted from brief picture descriptions (semantic coherence, syntactic complexity, pause patterns) effectively distinguished Alzheimer's disease from controls in multiethnic cohorts. Crucially, these acoustic and linguistic features reflect underlying neuropathology and may be less susceptible to cultural framing than explicit self‐reports, potentially providing culture‐neutral cognitive indicators.^4^ This approach does not seek to create a single, universally applicable SCD battery, but rather to identify objective biomarkers that can complement culturally informed clinical interpretation.

The findings by Farias et al. move the field beyond simply documenting prevalence differences and challenge assumptions about uniform SCD significance across populations. Future research must actively integrate cultural neuroscience perspectives, leverage advanced assessment technologies like AI for precision phenotyping, and translate these insights into culturally tailored public health initiatives and intervention strategies. Only by acknowledging and addressing this complexity can we ensure equitable progress in leveraging SCD for early detection and prevention of dementia across all communities. (Supporting Information).

## FUNDING

The authors have nothing to report.

## CONFLICTS OF INTEREST STATEMENT

The authors declare no conflicts of interest.

## Supporting information



Supporting Information: alz71141‐sup‐0001‐SuppMat.pdf
